# A cascading approach using se-resnext, resnet and feature pyramid network for kidney tumor segmentation

**DOI:** 10.1016/j.heliyon.2024.e38612

**Published:** 2024-09-27

**Authors:** Justice Kwame Appati, Isaac Adu Yirenkyi

**Affiliations:** Department of Computer Science, University of Ghana, Accra, Ghana

**Keywords:** SE-ResNeXt, Feature pyramid network, Segmentation, ResNet, Cascaded, Kidney tumor

## Abstract

Accurate segmentation of kidney tumors in CT images is very important in the diagnosis of kidney cancer. Automatic semantic segmentation of the kidney tumor has shown promising results towards developing advance surgical planning techniques in the treatment of kidney tumor. However, the relatively small size of kidney tumor volume in comparison to the overall kidney volume, and its irregular distribution and shape makes it difficult to accurately segment the tumors. In addressing this issue, we proposed a coarse to fine segmentation which leverages on transfer learning using SE-ResNeXt model for the initial segmentation and ResNet and Feature Pyramid Network for the final segmentation. The processes are related and the output of the initial results was used for the final training. We trained and evaluated our method on the KITS19 dataset and achieved a dice score of 0.7388 and Jaccard score 0.7321 for the final segmentation demonstrating promising results when compared to other approaches.

## 1 Introduction

Early diagnosis as well as accurate and precise determination of the extent of kidney tumor plays a significant role in the treatment strategies for patients with kidney tumors to prevent the spread of kidney related infection and undergoing a nephrectomy. The kidney organ is a vital organ which plays an essential role in the human body [1,2]. Kidney tumor is among the most lethal cancers in the world and the 13th most common cancer in the world, accounting for 2.4 % of all cancers, with more than 330,000 new cases diagnosed yearly [3]. Kidney tumors may either be benign or malignant. Benign tumors are tumors that remain confined to their original location and do not extend to affect other parts of the body. In contrast, malignant tumors spread rapidly and invade other sites of the body [4]. The precision of tumor segmentation affects the accuracy of radiotherapy treatment, as it gives detailed structural information into the anomalies for medical experts to assess and analyze critical clinical conditions [5]. It is therefore critical and a significant prerequisite to accurately segment the kidney tumor in order to localize the interest area and perform surgery to remove the tumor. In most medical centers, when a patient is diagnosed with a kidney tumor, the usual approach of segmenting the kidney tumor involves a traditional human-based manual segmentation, where medical experts need to analyze and examine the kidney's images to identify the tumor's specific area. However, manually segmenting kidney tumors is usually a challenging and time-consuming process, often leading to misinterpretation due to the subjective factors associated with the radiologist's expertise which can be costly [[6], [7], [8], [9]]. Human examination can be subjective and the outcome can change between practitioners, leading to a level of uncertainty in quantitative estimation. The traditional approach of manually segmenting kidney tumors is not only a time-consuming and labor-intensive process, but prone to resulting in inconsistent outcomes among specialists which would produce unsatisfactory results in clinical applications and errors [10]. As a result, there is the need to automatically segment kidney tumors so that medical specialists can use that information to perform surgery and reduce the risk of individuals undergoing kidney tumor surgery. Due to these numerous issues related to manual human-based segmentation approaches, Automated approaches are needed. Convolutional neural networks (CNNs) have been increasingly used in medical image analysis, including kidney tumor segmentation. CNNs are deep learning algorithms that can learn to identify complex image patterns, making them particularly effective for image segmentation tasks. They have achieved great success in medical image segmentation and detection tasks [11]. CNN has been used to segment different tumors in the body, such as segmentation of the brain [12], segmentation of the liver [13], segmentation of the lung [14], segmentation of the prostate [15], segmentation of the skin [16] and pancreas [17,18]. These methods use different algorithms and deep learning techniques to segment the kidney and identify tumor locations.

Currently different deep learning approaches are used to delineate kidney tumor without manual efforts. However, accurate segmentation of the kidney tumor is challenging due to the small size and shape of the tumor, unclear boundaries and the need to identify different features during segmentation. Training deep neural networks from scratch to extract the unique features in the dataset can be computationally expensive and time consuming and the model would end up not being able to learn all the necessary features which creates a gap where pretrained models can be used to transfer the knowledge gained from learning on large datasets in order accurately extract unique features during segmentation ad improve the performance. In this study we plan to solve this issue by proposing a two-stage cascaded automated approach leveraging on transfer learning using Squeeze and Excitation ResNeXT and ResNet embedded with a Feature Pyramid network to accurately segment and identify small size kidney tumors of while identifying the unique features in the images.

## 2 Related works

Recently, several works have been done using different convolutional neural network approaches to segment kidney tumors. In the study of Hou et al. [19], a triple-stage self-guided network for kidney tumor segmentation to solve the issue of small tumor sizes and difficulty distinguishing between tumors and cysts was proposed. The architecture consisted of three stages, the first of which involves obtaining the rough location of the kidney and tumor based on a low-resolution 3D U-Net from down-sampled 3D CT images. The Second stage involved using the output segmentation map from the first stage to input into a full-resolution 3D network to optimize and smooth the kidney boundary and obtain an initial tumor segmentation result. Finally, in the third stage, the tumor boundary was refined to retain false positives that may have been caused by cysts. But these approaches did not deeply considered the ratio of the kidney tumor region to the whole image which would create problems of sample inbalance. To address this, Xie, Li, Lian, Chen, & Luo [20], proposed a coarse-to-fine approach using SE-ResNeXT U-Net architecture which combined the advantages of Squeeze and Excitation, ResNeXT, and U-Net to address the issues of low contrast, irregular motion, diverse shapes, and sizes which makes the segmentation of kidney tumor task challenging. To utilize the context information and the key slices a two-stage coarse-to-fine manner was implemented where in the first stage the model was trained to perform a coarse segmentation in the whole images, and then, key slices are obtained by finding max contours. The output was then fed into the second stage to refine the segmentation and perform a fine segmentation to accurately segment the kidney tumors. The refinement network had the same architecture as the coarse segmentation architecture. In the study of Qayyum, Lalande, & Meriaudeau [7], a 3D residual network composed of various combinations of residual blocks was proposed. The proposed network uses SE blocks to capture spatial information based on the reweighting function in a 3D 1 RN which was evaluated on both on the Kidney Tumor Segmentation 2019 dataset and the public MICCAI 2017 Liver Tumor Segmentation dataset.

To improve on the results, Zhao, Chen, & Wang [21], proposed a coarse to fine framework based on the nnU-Net for the segmentation of the kidney and kidney tumor. The approach consisted of three major steps which are coarse segmentation to obtain the initial segmentation and crop the kidney region of interest. The second step which is the fine segmentation involves using nnU-Net to classify the kidney from the cropped kidney region of interest. In the third step two separate nnU-Nets are used to segment the kidney tumor and mass which was combined with the fine segmentation to obtain the final segmentation. Hsiao et al. [22], proposed an architecture which uses an EfficientNet-B5 as the encoder and a FPN to generate prediction layers at different scales with input from multi-scale feature layers as the decoder. This architecture helped to overcome the challenges of irregular shapes and inconsistent locations of pathological kidneys, which made it difficult to accurately segment the kidney area from the abdomen's CT images. The FPN allows the model to capture context at multiple scales, which is crucial for accurately segmenting complex structures like the kidneys. The proposed method uses a framework consisting of four stages of window selection, model selection, loss function selection, and data augmentation to determine the appropriate hyper-parameters and model structure. This ensures that the model is optimized for the specific task of kidney segmentation and can perform well under different conditions such as varying voxel spacing, anatomical planes, and kidney and tumor volumes. To address the challenge of the long-range dependencies of feature maps being overlooked, Wen, Li, Shen, Zheng, & Zheng [23], proposed a segmentation network called SeResUNet. The proposed SeResUNet consisted of an encoder-decoder architecture, where the encoder uses a squeeze and excitation module to learn high-level semantic features and capture the long-range dependencies between different channels of the feature maps. Specifically, the encoder adopts the SE-ResNet50 architecture, a residual network with a squeeze-and-excitation block added after each residual block. The decoder uses the U-Net architecture and is connected to the encoder through skip connections for feature concatenation. To further improve the accuracy and stability of the network, multi-level deep supervision in the decode was introduced, where deep supervision is performed on each layer of the decoder so that the shallow layer can be fully trained. In addition, to estimate the degree of inconsistency between the predicted segmentation map of the model and the ground truth, a weighted cross-entropy loss function was used. He, Zhang, Pei, & Huang [24], proposed an approach involving a two-stage cascaded deep neural network with multi-decoding paths for kidney tumor segmentation. The study aimed to address the challenges of precise segmentation of tumors and cysts, which have different morphologies with blurred edges and unpredictable positions. To overcome these difficulties, the authors proposed a cascaded deep neural network that first accurately locates the kidney area through a 2D U-Net and then segments the kidneys, kidney tumors, and renal cysts through a multi-decoding segmentation network from the kidney. The proposed model consisted of two stages. In the first stage, the kidney localization network is composed of an encoding path with four encoder blocks. The kidney localization network aims to preprocess the image to locate the kidney through 2D U-Net to obtain an accurate kidney area, then use the kidney area as the bounding box of the original CT, crop to get the input image, and train the MSD-Net to segment kidney, tumors, and cysts.

However, these approaches did not considered the unique features of the images during feature extraction and to address that, Feng, Kou, Tang, & Li [25], proposed a cascading segmentation network model for kidney tumor segmentation to address the challenges of small size and irregular distribution of tumors. The model was designed as a two-step process with a localization network in the first step to scan the kidney and identify slices containing tumors through the coarse segmentation network. The YOLO-V5 network was used for the coarse network. The second step employs a segmentation framework embedded with a FPN module for finely segmented kidney tumors. One of the main advantages of this approach is that it helped to strengthen the feature conduction of the entire network and alleviate the gradient disappearance problem. Additionally, the feature reuse is improved, which enhances the learning ability of the network.

## 3 Methods and materials

**Fig. 1:**
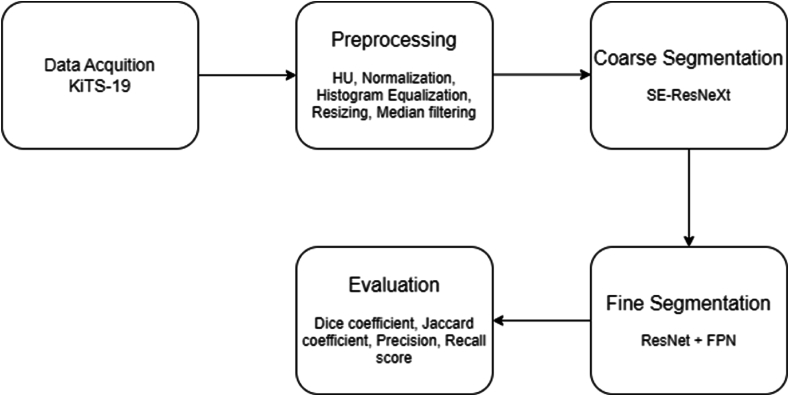
Outline of the methodology.

In this study the approach used to segment kidney tumors involved acquiring the dataset, preprocessing the dataset, performing a coarse segmentation to roughly segment the tumor, performing a fine segmentation to precisely delineate the kidney tumor and evaluating the performance metrics of the approach as illustrated in Fig. 1.

### 3.1 Dataset

The dataset used in this paper is the 2019 Kidney Tumor Segmentation Challenge dataset (KiTS19) [26]. The dataset consists of a collection of segmented CT imaging and treatment outcomes for 300 patients who were diagnosed and treated with either partial or radical nephrectomy between the period of 2010 and 2018. Gathering the dataset from each patient went through four main steps to ensure that the right CT imaging was used for practical purposes. These tasks were carried out by experienced urologic oncologists who specialized in kidney tumors. 210 of the scans were used for training and the remaining 90 were used for testing.

### 3.2 Preprocessing

The dataset was preprocessed to enhance the performance of the model. Various preprocessing techniques including Hounsfield unit windowing, resizing, histogram equalization, median filtering and normalization were applied to make the dataset ready for training. The range of kidneys was chosen to be [−200, 500] and a window and a level was chosen to adjust the display of the image. Selecting an appropriate range of values was important to target only the organ of interest in the segmentation process. Given the window level and window width, the maximum and minimum HU values representing the upper and lower limits of HU values were specified for the window using Equation (1) and Equation (2). To minimize the use of memory and the demand of high computational power, the slices were resized from 512 × 512 pixel to 256 × 256 pixel to ensuring that all images have a consistent dimension while retaining the essential features of the original image. A median filter with a kernel of size 3 × 3 was used to reduce the noise and any other unwanted artefacts while preserving the edges. All the slices were extracted from the scans and converted to a PNG format to facilitate easy processing. Since the focus of the segmentation was the tumor, only the tumor was seen as the foreground while all other parts were seen to be background.

(1) $$\begin{array}{c} \text{minimum}value=window\;level-\frac{widow\;width}{2}\; \end{array}$$

(2) $$\begin{array}{c} maximum\;value=windowlevel+\frac{window\;width\;}{2}\; \end{array}$$

### 3.3 Metrics evaluation

To evaluate the model's performance in this study, the metrics considered in this study were the Dice coefficient, Jaccard coefficient, Precision and Recall value which are shown in Equations (3), (4), (5), (6) respectively

(3) $$\begin{array}{c} Dice\;score=\frac{2\times \left|\left(x\cap y\right)\right|}{\left|x\;\right|+\left|y\right|} \end{array}$$

(4) $$\begin{array}{c} Jaccard\;coefficient=\frac{x\cap y}{x\cup y} \end{array}$$

(5) $$\begin{array}{c} Precision=\frac{TP}{TP+FP} \end{array}$$

(6) $$\begin{array}{c} Recall=\frac{TP}{TP+FN\;}\; \end{array}$$

Where x is the predicted data, y is the label data, TP is number of correctly predicted positive outcomes, FP is the number of incorrectly predicted positive outcomes and FN is the number of positive outcomes incorrectly predicted.

### 3.4 Proposed architecture

The proposed approach which combines the advantages of transfer learning of the SE-ResNeXt, ResNet and FPN as shown in Fig. 1 is implemented in two stages where the tumor region was first extracted and implemented as the initial segmentation and the results was then used for the final segmentation to accurately delineate the kidney region.

### 3.5 Coarse segmentation

**Fig. 2:**
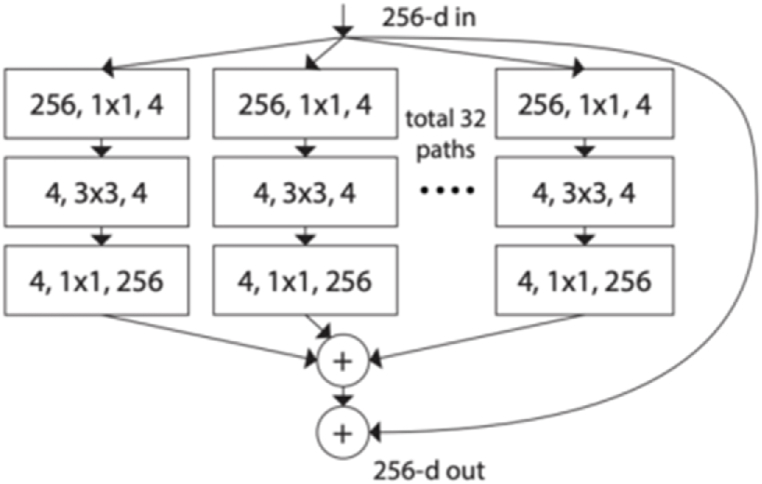
A ResNeXt block with cardinality of 32.

The coarse segmentation of the cascaded approach was implemented using Squeeze and Excitation Network [27] and ResNeXt [28] mostly know as (SE-ResNeXt) architecture. The ResNeXt is a combination of ResNet [29] and Inception [30] which has shown remarkable performance in computer vision tasks. ResNeXt uses cardinality to improve the learning of the network allowing different paths to have different numbers of filters. Fig. 2 shows a ResNeXt block with cardinality of 32 with the same number of input channels, filer size and output channels.

**Fig. 3:**
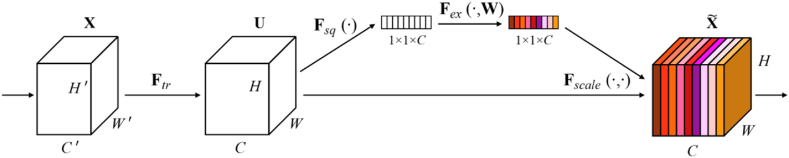
A Squeeze Excitation block.

To further improve the network Squeeze-and-Excitation (SE) modules were integrated into each residual block. The SE modules introduced into the residual blocks helps to improve feature recalibration and channel-wise adaptively in the network. As a result, the network can prioritize more informative features while suppressing less relevant information, enabling a more focused and effective learning process. Fig. 3 illustrates the architecture of the SE building block, featuring a transformation F_tr_ that corresponds to the mapping of the input X to the feature maps U.

The initial convolutional layer had an input layer of one representing a grayscale with one color channel with an output channel of 32 was used during the initial convolution. After the first convolution, batch normalization was implemented to accelerate learning, reduce overfitting and standardize the activations of the convolutional layer across the entire batch of input data. This was computed by calculating the mean and variance of the small batch, normalizing the batch and applying a scale and shifting the normalized values as expressed in Equation (7). This is applied to the neuron's output just before applying the activation function. Mathematically the Batch Normalization can be expressed as;

(7) $$\begin{array}{c} y_{i}=\left(\frac{x_{i}-m_{b}}{\sqrt{\sigma_{b}^{2}+e}}\right)\times \gamma +\beta \; \end{array}$$

Where y_i_ is the output of the Batch Normalization, x_i_ is the input to the layer, m_b_ is the mean of the mini batch, e is a small constant for stability, $\sigma_{b}^{2}$ is the variance of the mini-batch. and γ+β are the learning rate to shift and scale the mean and standard deviation respectively.

Max-Pooling was then applied in the initial layers to down sample the feature maps and reduces the spatial dimension. A 3 × 3 pooling window with a stride of 2 and a padding of 1 was used. Four series of Residual blocks was added with each containing convolutional layers with a batch normalization and a ReLU activations to help the network learn hierarchical features. In each of the residual blocks, a skip connection was used to allow the flow of gradient more easily through to the network. A Global Average Pooling layer was then added to compute the spatial average of the feature maps which helped to reduce the spatial dimensions to a single vector. Finally, a fully connected layer was added to return the outcome of the network.

### 3.6 Fine segmentation

**Fig. 4:**
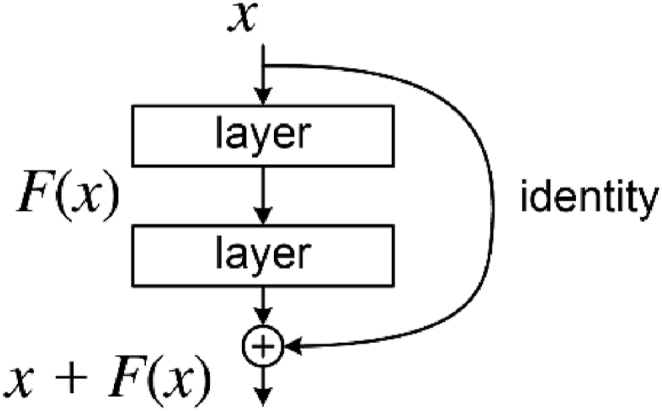
A ResNet block.

The second model within the cascaded approach integrates a pre-trained ResNet, complemented by an FPN as the connecting structure. The ResNet as shown in Fig. 4 acts as the backbone network to extract features from the input image and produce a feature map at the different levels of resolution. ResNet which is pre-trained on a large dataset which offers great feature extraction and a good starting point for the model to learn through transfer learning. The transfer learning inculpated in this architecture helps the model generalize well on the tumor segmentation, speeding up training significantly and helping to achieve competitive performance with small datasets. The FPN [31] is used as the neck in this architecture to fuse the features from all the levels to enhance features with both higher accuracy and richer semantics. To address the challenge of multi-scale object recognition, the FPN has multiple layers, each with different spatial resolutions and semantic information. Higher levels of the pyramid have lower spatial resolution but richer semantic information, while lower levels have higher spatial resolution but less semantic information. It uses lateral connections that connect feature maps from the top-down pathway that allows high level semantics from the top layers to be combined with the detailed spatial information from the lower layers. The FPN combines the high- and low-resolution feature maps to enhance the feature with both accurate spatial information and rich semantics.

The architecture combines the features of ResNet functioning as the backbone and FPN operating as the neck with a segmentation head for segmentation. Five ResNet convolutional layers, an average pooling layer, a fully connected layer and a SoftMax were used. Each of the convolutional layer had different number of residual blocks with a bottleneck structure which was the basic building block. The initial convolutional layer used a 3 × 3 filter, a stride of 2 × 2 and a padding of 3 × 3 with a batch normalization and ReLU activation. Skip connection in the form of identity mappings which connected the input directly to the output of the block was introduced to propagate through the network without passing through many non-linear activation functions which helped to alleviate the problem of vanishing gradients.

**Fig. 5:**
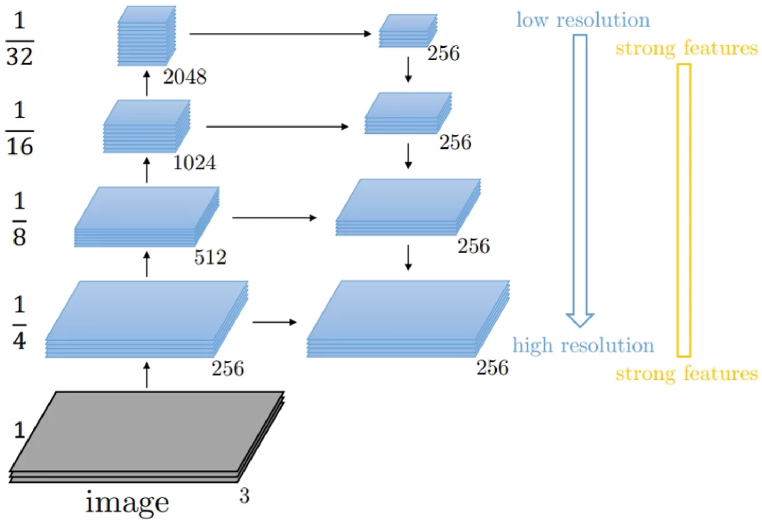
An FPN block.

After the ResNet layers, the architecture includes an FPN component, which helped in handling multi-scale features. It has a top-down pathway which up-sample feature maps to a higher spatial resolution and lateral connections which allow high level semantic from the top layers to be combined with detailed spatial information from the lower layers to build a pyramid of feature maps as shown in Fig. 5. This helps to recover spatial information. It consists of four layers with FPN blocks and a max-pooling which receive a feature map to generate features. Each layer is an instance of the FPN block and helps for generating features at different pyramid levels and fusing the features helping to facilitate the extraction of multi-scaled features, enabling better performance in the training. (Code is made available: https://github.com/Data-Intel-and-Swarm-Analytics-Lab/Cascaded-approach-using-SE-ResNeXt-ResNet-and-Feature-Pyramid-Network-for-kidney-tumor-segmentation). The model was trained using Core i7 HP Pavilion Gaming Laptop 16 GB RAM with NVidia GeForce GTX 1660 Ti having 6 GB dedicated graphics.

**Table:** Ablation Study

In the study of [20], a coarse-to-fine approach was utilized using a proposed SERU model. In the first stage the SERU model was trained and produced the coarse segmentation results. In the second stage, the same SERU model with different input scale were used to perform the fine segmentation. In their approach the same model which was used to train the coarse was also utilized in the fine segmentation.	In the study of [32], a two-stage cascade network utilizing a coarse-to-fine was used to segment. In the first stage, a modified 3D U-Net was used. In the second stage, a model called Masses-Net was designed to perform the fine segmentation utilizing a multi-dimension feature to learn more spatial and contextual information obtaining a dice score of 0.563.	In our study, we implemented the coarse segmentation of the cascaded approach using Squeeze and Excitation Network and ResNet. In our second model, we integrated a pre-trained ResNet, complemented by an FPN as the connecting structure. The pre-trained helps the model to generalize well on the tumor segmentation, speeding up training significantly and helping to achieve competitive performance. Even though all these approaches utilize a coarse-to-fine approach, our approach integrates pre-trained models improving the generalization on the tumor segmentation. The strength of the SE-ResNeXt, ResNet, and FPN models to accurately segment the kidney tumor. Also, different models were used in both the coarse and fine stage to enhance the performance

## 4 Results and discussion

### 4.1 Preprocessing results

**Fig. 6:**
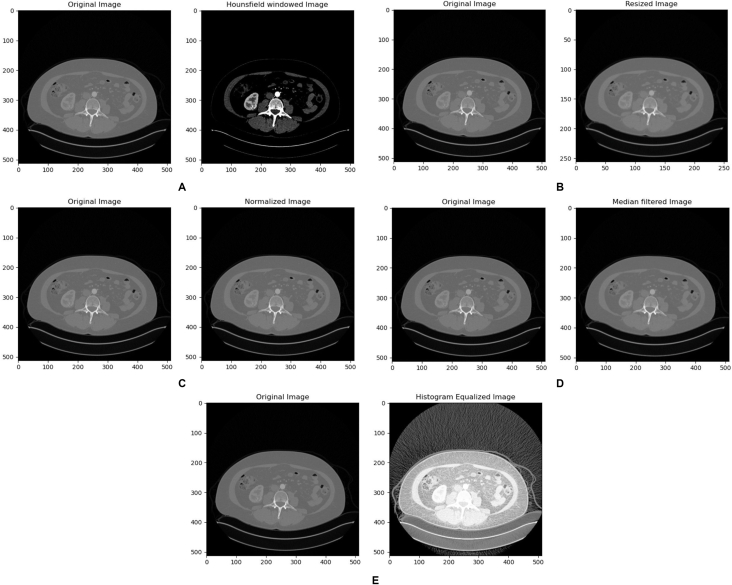
Results of Preprocessing techniques.

In this study five preprocessing techniques were applied to enhance the quality of the images before training to improve the model's ability to segment the tumors. Fig. 6(A) shows an image before and after applying HU windowing technique which yielded good results in enhancing the quality of the image. By carefully selecting the window width and level, the images were effectively processed to focus on small anatomical details and features, and neglect the fatty tissues and all the other tissues which do not have the radio-density within that range of window leading to clearer and more visible representations for the model to detect and segment. Fig. 6(B) shows an original and resized image that has undergone the resizing process of changing the pixel dimension from 512 × 512 pixel to 256 × 256 pixel to optimize image storage requirements and computational processing efficiency without compromising on the essential features in the image. Fig. 6(C) shows the results before and after applying normalization to the image which helped to align the intensity values of the image with a standardized scale, thereby facilitating the segmentation algorithm's outcome. This technique helped to enhance the quality of the images and as a result improve the accuracy of the segmentation model. Fig. 6(D) shows the results of a median filtered image which proved to be necessary in enhancing the quality of the image while eliminating high-frequency disturbances, contributing to enhanced segmentation accuracy and outcome. The improved image quality resulting from the noise suppression contributed to the extraction of more accurate feature representations and structural elements. Fig. 6(E) shows an image before and after applying the histogram equalization which played a critical role in improving the perceptual quality and contrast characteristics of the image, thereby supporting the subsequent segmentation process. This aspect facilitated a more precise differentiation between various image regions, enabling the segmentation algorithm to identify distinct boundaries and features with greater accuracy.

### 4.2 Segmentation results and discussion

In the first experiment all the masks in the dataset were converted to have only the tumor excluding the kidney. So, the tumor was the foreground, and all other parts were seen as the background. This experiment used all the datasets to train the model including tumor and non-tumor images in the dataset. This involved a cascaded approach where a coarse segmentation was performed first to do initial segmentation of the tumors before a final segmentation to accurately segment the tumors in the kidney.

**Fig. 7:**
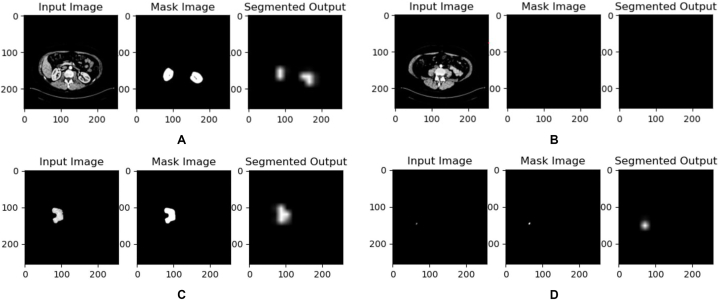
Results of experiment I.

The proposed model successfully achieved its objective of effectively segmenting large, medium and small-sized kidney tumors as shown in Fig. 7(A)–. (C) and (D). The model's capacity to detect the absence of tumors as shown in Fig. 7(B) is a promising indication of its proficiency in distinguishing healthy kidney tissue from tumorous regions, thereby bolstering its reliability for diagnostic applications. The evaluation metrics which included the Dice score, Jaccard score, precision score, and recall value, were measured and used to assess the performance of the segmentation model. A dice score of 0.7388 and a Jaccard score of 0.7231 were recorded as the best model over the training period indicating the model's ability to accurately identify and delineate kidney tumors within the CT kidney tumor dataset.

The successful segmentation of kidney tumors, which achieved the objective of this research of identifying small size kidney tumors showed the pivotal role played by the pre-trained ResNet model in the segmentation framework. Leveraging a pre-trained ResNet model helped to extract complex hierarchical features from kidney tumor dataset. This transfer learning approach promoted the enhancement of the segmentation model's overall performance, particularly in capturing the small features tumors in the kidney. Furthermore, the pre-trained ResNet empowered the model with an inherent understanding of intricate imaging features, enabling it to achieve enhanced generalization and improved accuracy in tumor segmentation tasks, thereby contributing to more robust and reliable clinical decision-making processes. In addition to the pre-trained ResNet, the incorporation of the FPN further augments the segmentation model's capabilities, particularly in accurately delineating kidney tumors of various sizes. The FPN architecture, renowned for its multiscale feature representation, fosters a comprehensive understanding of the complex spatial hierarchy inherent in kidney tumor imaging data. By exploiting the hierarchical feature maps generated at multiple scales, the FPN framework facilitated the precise localization and segmentation of tumors, irrespective of their size variations.

Reducing the learning rate by 0.1 after every 5 epochs during the training process to fine-tune the model's optimization process had crucial implications for the overall performance and convergence of the segmentation model enabling more precise weight adjustments and facilitating smoother convergence to an optimal solution. The reduction in the learning rate helped to improve the model's stability and robustness, enabling a more gradual and controlled descent along the loss landscape. This helped the model to become less prone to overshooting the optimal solution, thereby minimizing the risk of oscillations and erratic behavior during the training process. The gradual reduction in the learning rate enhanced generalization and adaptability, enabling the model to capture kidney tumors within the data without overfitting which helped in accurate identification of diverse tumor sizes. The evaluation of the segmentation model using the SE-ResNeXt, ResNet, and FPN framework, as evidenced by the recorded metrics, showed the efficacy of the proposed methodology in achieving the study's key objectives.

In the second experiment, only kidney containing tumors in the dataset were used to train the model. A first coarse segmentation was done using the SE-ResNeXt followed by a second segmentation to finely segment the kidney tumors using ResNet embedded with the FPN.

**Fig. 8:**
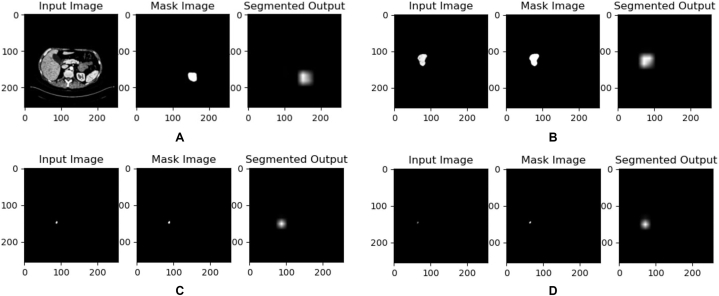
Results of experiment II.

The segmentation results revealed the progressive improvement in the model's performance, as evidenced by the incremental enhancement in various evaluation metrics, such as the Dice score, Jaccard score, precision score, and recall value, over the training epochs. In the final training, the best model saved which was used for inference had a dice score of 0.6926, a Jaccard score of 0.5297, a precision score of 0.6211 and a recall value of 0.7831. The model exhibited the capability to detect kidney tumors of varying sizes as evidenced in Fig. 8, showing promising results. Fig. 8(A) and (B) shows the effectiveness of the model in segmenting medium size tumors while Fig. 8(C) and (D) shows the outcome when there is very small or no tumor. The scored observed across dice score, Jaccard score, precision score and recall score affirm the effectiveness of the implemented approach in addressing the study's core objectives of the identification of small-size kidney tumors.

The findings of this research study demonstrated that the proposed model achieved higher scores across various evaluation metrics compared to the results of other works. The model's proficiency in identifying small size tumors and distinguishing healthy kidney tissue from tumorous regions, and its reliability in detecting the absence of tumors were among the key factors contributing to its superior performance. In the studies by Refs. [33,34], 210 samples from the KITs-19 dataset were used, whereas our dataset consisted of all the 300 samples from the KITs-19 dataset. Additionally [35], used a different dataset for their experiment which could account for the differences in results. The superior performance of the proposed model, as evidenced by the comparative analysis in Table 1, shows its potential as a promising solution for enhancing the accuracy and reliability of kidney tumor segmentation in clinical practice. The findings suggest that the integrated approach employed in this study can serve as a benchmark for future research endeavors aimed at advancing the field of kidney tumor segmentation and facilitating more informed decision-making processes in clinical settings.

**Table 1:** Comparison to existing work.

Approaches used	Dice Score	Jaccard Score	Precision Score	Recall score
[36]	0.6738	–	–	–
[37]	0.570	–	–	–
[38]	0.601	–	–	–
[39]	0.7300	–	–	–
[40]	0.384	–	–	–
[38]	0.601	–	–	–
[41]	0.5440	0.9857	0.5952	–
[33]	0.865	–	–	–
[42]	0.869	–	–	–
[43]	0.859	–	–	0.862
Experiment II	0.6926	0.5297	0.6211	0.7831
Experiment I	0.7388	0.7321	0.8662	0.8145

## 5 Conclusion

This study of segmenting kidney tumors has presented a novel and effective approach for the segmentation of kidney tumors. By using advanced deep learning architectures, including SE-ResNeXt, ResNet, and FPN, the proposed approach has demonstrated significant results in segmenting kidney tumors, enabling precise and reliable identification of tumors of varying sizes.

Furthermore, the comprehensive utilization of the SE-ResNeXt, ResNet, and FPN architecture, coupled with the optimized learning rate adjustment, played an important role in elevating the model's segmentation accuracy. By effectively leveraging the pre-trained ResNet model and the FPN, the proposed methodology demonstrated improved adaptability and robustness in capturing intricate tumor characteristics and delineating precise tumor boundaries.

The application of a coarse to fine segmentation approach to segment the tumors helped to accurately delineate the tumor boundaries and important features which produced great results helping achieve the objectives of this study. The successful integration of transfer learning using pre-trained ResNet models and FPN architecture has further enhanced the method's adaptability and generalizability, facilitating consistent and reliable segmentation outcomes which was able to achieve the objects of this study. The results were compared to existing approaches used to segment the kidney tumor with this approached. The findings of this research thesis contribute valuable insights and advancements to the field of kidney tumor segmentation, showing the potential of deep learning techniques to revolutionize clinical practices and enhance patient care in the domain of medical image analysis and kidney tumor segmentation.

However, certain avenues for future exploration and refinement remain unexplored. One crucial direction for future work involves the expansion of the experiments to encompass a more comprehensive analysis and evaluation of the proposed methodology. Leveraging additional experiments and validation on diverse datasets can further bolster the accuracy and performance of the segmentation model, thereby enhancing its adaptability and reliability in real-world clinical applications.

## Data availability

The data for the KITS19 is available using this link: https://github.com/neheller/kits19.

## CRediT authorship contribution statement

**Justice Kwame Appati:** Writing – review & editing, Supervision, Software, Resources, Project administration, Methodology, Investigation, Formal analysis, Data curation, Conceptualization. **Isaac Adu Yirenkyi:** Writing – original draft, Methodology, Investigation, Conceptualization.

## Declaration of competing interest

The authors declare that they have no known competing financial interests or personal relationships that could have appeared to influence the work reported in this paper.
